# Author Correction: Satellite mapping reveals extensive industrial activity at sea

**DOI:** 10.1038/s41586-024-07123-7

**Published:** 2024-02-02

**Authors:** Fernando S. Paolo, David Kroodsma, Jennifer Raynor, Tim Hochberg, Pete Davis, Jesse Cleary, Luca Marsaglia, Sara Orofino, Christian Thomas, Patrick Halpin

**Affiliations:** 1 Global Fishing Watch, Washington, DC USA; 2 Forest and Wildlife Ecology Department, University of Wisconsin–Madison, Madison, WI USA; 3 Marine Geospatial Ecology Lab, Nicholas School of the Environment, Duke University, Durham, NC USA; 4 Bren School of Environmental Science and Management, University of California, Santa Barbara, Santa Barbara, CA USA; 5 SkyTruth, Shepherdstown, WV USA

**Keywords:** Environmental impact, Energy infrastructure

Correction to: *Nature* 10.1038/s41586-023-06825-8 Published online 3 January 2024

In the version of this article initially published, Fernando S. Paolo was presented in the author list without a middle initial. Also, in the “SAR and AIS integration” section of Methods, in the sentence “To determine the optimal score, we estimated the total number of vessels with AIS devices that should have appeared in the scenes globally by summing *R*(length, spacing)*L*_match_ for all scenes”, “*L*_match_” should have been “*L*_inside_”. The errors have been corrected in the HTML and PDF versions of the article.

